# Quality of Life and Self-Determination: Youth with Chronic Health Conditions Make the Connection

**DOI:** 10.1007/s11482-014-9382-7

**Published:** 2015-01-07

**Authors:** Janette McDougall, Patricia Baldwin, Jan Evans, Megan Nichols, Cole Etherington, Virginia Wright

**Affiliations:** 1 Thames Valley Children’s Centre, 779 Base Line Road East, London, ON Canada N6C5Y6; 2 Social Science Center, Department of Sociology, Western University, London, ON Canada N6A 5C2; 3 Bloorview Research Institute, 150 Kilgour Road, Toronto, ON Canada M4G 1R8

**Keywords:** Youth, Chronic conditions, Quality of life, Self-determination, Qualitative research

## Abstract

While optimizing quality of life (QOL) is a key goal of rehabilitation care, no previous study has reported on what ‘QOL’ means to youth with chronic health conditions. In addition, no qualitative studies have explored the relationship between QOL and self-determination (SD). Objectives of this qualitative study were to examine: what the terms ‘quality of life’ and ‘self-determination’ mean to youth with chronic conditions; the factors these youth think are linked with these concepts; the relationship they see between concepts, the types of future goals youth have and how they view the connection between their SD and these goals. A descriptive methodology was used. A purposive sample of 15 youth aged 15 to 20 years was obtained. Youth had cerebral palsy, a central nervous system disorder, or autism spectrum disorder. Semi-structured interviews were conducted first, followed by a focus group. Line-by-line coding of transcripts was completed, codes were collapsed into categories, and themes identified. Participants viewed QOL as an overarching personal evaluation of their life, and used terms such as satisfaction and happiness to describe the concept. Factors related to QOL included: ‘relationships’, ‘supportive environments’, ‘doing things’, ‘personal growth and moving forward’, and ‘understanding of self/acceptance of disability’. Participants described SD in such terms as confidence and motivation. Contributors to SD were: ‘personal strengths’, ‘interdependence’, and ‘functional independence’. SD was considered important to QOL. Youth goals were reflective of the goals of most adolescents. They identified the importance of having key goals that were of personal interest to them. This study adds consumer-based information to the debate over the meaning of QOL. Service providers and decision makers should be aware of the factors that youth feel impact their QOL and SD, the importance of SD to youth QOL, and of SD to future goals, and consider this information when tailoring therapeutic interventions.

## Introduction

The number of children and youth living with a chronic condition has increased over the last decades with advances in medical treatments (Newacheck et al. 2006). Yet, few cures for these conditions have been found, often leaving individuals with condition-, treatment-, or environmentally-related disabilities (van der Lee et al. 2007). Over the past 30 years, quality of life (QOL) has increasingly become a key focus of health and rehabilitation care and research (Moons 2006). This enhanced emphasis has been attributed to a shift away from a medical model that expects that scientific, biomedical, and technological advances will directly lead to cures and extended life years, towards a biopsychosocial or holistic model that also emphasizes additional influences on life quality, such as personal, family, and community factors, and recognizes the complex interrelationship among all of these factors (Verdugo et al. 2005). The increased interest in QOL has also been linked with the rise of the consumer empowerment movement with its emphasis on person-centered service delivery, valued outcomes, and self-determination (Verdugo et al. 2005).

However, the study of QOL in the area of health and rehabilitation has not been without challenges. A primary concern has been the lack of clarity about what the concept means. No consensus has emerged regarding its definition, conceptualization, or measurement. Some argue that QOL is an entirely subjective and holistic assessment of life overall (Moons 2006), while others see it as multi-dimensional, and comprised of numerous subjective and objective domains of life (Verdugo et al. 2005). QOL is often used interchangeably with other concepts such as health-related QOL (HRQOL), happiness, life satisfaction, or well-being. Opinions about the worth of the QOL concept range from the standpoint that it is equivocal (Gutenbrunner et al. 2011), extends beyond the realm of what health and rehabilitation services should be expected to address (Meyer et al. 2011), and may not be measureable (Tesio 2009), to the view that it is a primary goal and outcome of health and rehabilitation services (Anderson and Burckhardt 1998; King et al. 2002). Despite ongoing debate, focus on QOL is now widely present in the literature and is generally accepted as a legitimate area of health and rehabilitation research and an important outcome of clinical practice. Consequently, continued study of how to define and measure this concept has strong relevance with regard to helping to move the field forward.

QOL conceptual frameworks and measurement tools used within health and rehabilitation may contain any number of indicators or distinct domains. An analysis of the literature on individual-referenced QOL by Schalock (2004) yielded 125 indicators that could be subsumed under eight core QOL domains: physical well-being, emotional well-being, personal development, self-determination, interpersonal relations, social inclusion, material well-being, and rights. While most QOL/HRQOL questionnaires have been developed for adults with chronic conditions, a number have also been created for children and youth with chronic conditions (Detmar et al. 2006). However, most of these pediatric QOL measures have been developed based on “expert” and/or parental opinion with little to no input from children and youth (Detmar et al. 2006). This is unfortunate as research has indicated that children as young as 5 years can competently report on their health and disability (Rebok et al. 2001; Young et al. 1996). Thus, rather than relying on expert opinion or proxy reports, it is both possible and essential that QOL/HRQOL questionnaires and studies focus on personal definitions and descriptions of QOL generated by children and youth themselves (McEwan et al. 2004).

This perspective gap coupled with the possibility for direct child and youth input has been recognized, and over the last 15 years, a number of qualitative studies (Berntsson et al. 2007; Davis et al. 2008; Foley et al. 2012; Hill et al. 2014; Nicholas et al. 2007; McEwan et al. 2003; Panepinto et al. 2012; Parkinson et al. 2011; Ronan et al. 1999; Shikako-Thomas et al. 2009; Skjerning et al. 2014; Squitieri et al. 2013; Vinson et al. 2010; Young et al. 2007) have been conducted with children and youth with various chronic conditions to explore the factors that they identify as important to their QOL. The primary themes/domains/factors identified in these studies are listed in Table 1.

**Table 1:** Review of qualitative studies examining QOL for children and youth with chronic conditions

Study	Year	Condition/disability	Sample size	Age range	Concept explored	Themes/domains/factors
Ronan et al.	1999	Epilepsy	29	6–10	HRQOL	Experience of epilepsy, life fulfillment and time use, social issues, impact of epilepsy, attribution
McEwan et al.	2004	Epilepsy	22	12–18	QOL and psychosocialdevelopment	Peer acceptance, development of autonomy, school-related issues, epilepsy as part of me, future
Berntsson et al.	2007	Long-term illness or disability (e.g., postcerebral tumour, cystic fibrosis, heart disease)	15	12–19	Well-being	Feeling of acceptance/disability as a natural part of life, feeling of support, feeling of personal growth
Nicholas et al.	2007	Inflammatory bowel disease	80	7–19	HRQOL	Treatment and symptom concerns, vulnerability and lack of control, negative self-perception relative to peers, benefits of social support, personal resources in coping
Young et al.	2007	Cerebral palsy	28	8–13	HRQOL	Social relationships, home and school environment, self and body, recreational activities and resources, relationships with family members other than parents, inclusion and fairness, home life and neighborhood, pain and discomfort, environmental accommodation of needs
Davis et al.	2008	Cerebral palsy	17	13–18	QOL	Physical health and physical changes, functioning, pain and discomfort, communication, social well-being and acceptance, relationships and sexuality, participation, independence and transitioning, emotional wellbeing, and self-esteem, acceptance of disability, supportive physical environment and equipment, getting on well at school
Shikako-Thomas et al.	2009	Cerebral palsy	12	12–16	QOL	Intrinsic strengths (managing challenges, self-perceptions, self-advocacy, mastery, aspects of cerebral palsy (severity, aging with a disability), family, peers, school, community, perceptions of others, pursuing preferences in the face of obstacles
Vinson et al.	2010	Cerebral palsy	41	6–12	QOL	Family, friends, pets, hobbies, physical play, physical health/physical needs, school/education, religion, electronics, entertainment, travel
Parkinson et al.	2011	Cerebral palsy	28	8–13	HRQOL	Social relationships, home environment, school environment, self and body, recreational activities and resources
Foley et al.	2012	Disability (e.g., cerebral palsy, autism spectrum disorder, Down syndrome)	20	8–16	Well-being	Having things to do, good friends, home and family life, anxiety about school performance, managing life issues (coping with peers, striving toward goals, developing skills), feeling good about oneself
Panepinto et al.	2012	Sickle cell disease	13	5–18	HRQOL	Pain (severity, limiting activities, fear), fatigue (affects school, work, sleep) emotions, symptoms and treatment, social relationships, communication with peers, healthcare providers
Squitieri et al.	2013	Neonatal brachial plexus palsy	18	10–17	QOL	Social impact and peer acceptance, emotional adjustment and psychological coping, body image concerns, functional limitations, physical and occupational therapy, pain, family dynamics
Hill et al.	2014	Osteogenesis imperfecta	10	6–17	HRQOL	Keeping safe, reduced function, pain, fear, independence, isolation/being different
Skjerning et al.	2014	Celiac disease	23	8–18	HRQOL	Symptoms, diagnosis process, self-perception, social and emotional impact of disease, thoughts about future, coping with food, coping with social situations

*QOL* quality of life, *HRQOL* health-related quality of life

These studies have provided a critical initial understanding of what is important to or what factors contribute to the QOL of children and youth with chronic conditions. This information is useful to service providers for knowing what aspects of life to focus on when working toward enhancing youth QOL. It can also serve to guide the development of measures to assess those aspects of life that contribute to overall life quality. However, it does not get any closer to understanding what the concept of QOL actually means to youth. Indeed, all of the studies conducted so far have asked questions that only reveal the factors that contribute to QOL or HRQOL such as: “Can you tell me what is important for you to feel good in everyday life” (Berntsson et al. 2007), “What do you think is important to your QOL?” (Davis et al. 2008), or “How does epilepsy affect your day-to-day life” (Ronan et al. 1999). There seems to be an underlying assumption that QOL is multidimensional, and comprised of various domains, that need to be identified. In both a clinical and a research context, this may be problematic, as QOL may be conceptualized and measured as simply the product of specific aspects of life that do not necessarily capture or represent the essence of the concept.

To our knowledge, while studies have been conducted to report on what the term QOL means to youth without chronic conditions and with survivors of childhood illness (see discussion section) no previous study has done this with youth who are living with a chronic condition. This information is important for knowing whether these youth define the concept as a subjective, holistic assessment of life overall, as multi-dimensional, and comprised of numerous subjective and objective domains of life, or as something else altogether. Research that inquires about the exact definition of QOL from the perspectives of the individuals being studied can contribute to important client-based knowledge to the ongoing debate of the meaning of this concept. By extension, it could also provide insight into what QOL measurement approach best reflects the perspectives of persons with chronic conditions, in this case, youth.

Moreover, although a number of previous qualitative studies (Skjerning et al. 2014; Berntsson et al. 2007; Davis et al. 2008; Foley et al. 2012; Hill et al. 2014; McEwan et al. 2003; Shikako-Thomas et al. 2009) have identified concepts associated with self-determination (SD) (e.g., autonomy, personal growth, goal orientation, mastery, independence, self-advocacy, future focus) as being important to QOL for youth with chronic conditions, none have inquired further about the meaning or importance of SD to these youth. Indeed, no qualitative studies were found that inquire about SD from the viewpoint of young people with chronic conditions. Such an exploration is relevant since several quantitative studies have indicated a significant positive relationship between SD and QOL for youth and young adults with chronic conditions (Lachapelle et al. 2005; McDougall et al. 2010a; Nota et al. 2011; Wehmeyer and Schwartz 1998) For example, Nota et al. (2011) found significant correlations between level of SD and higher quality of life in 1400 adolescents with chronic conditions. Moreover, Wehmeyer (2005) has posited SD to be the core indicator of quality of life. Further, SD is considered to play a crucial role in the successful transition from adolescence to adulthood (Nota et al. 2011). Gaining a greater understanding of how youth with chronic conditions define SD, what they think contributes to their SD, how they view the relationship between SD and QOL, and the connection they see between being self-determined and achieving future goals would offer insight into how families, service providers, and society in general can support youth to take personal control of their lives and future as they transition to early adulthood.

### Purpose

The purpose of this research was to extend on past qualitative studies that have examined the factors that are important to the QOL of youth with chronic conditions. Specific objectives that were addressed in this paper included: (a) understanding what the concepts of QOL and SD mean to these youth; (b) further exploring the factors related to their QOL as well as contributors to their SD; c) investigating whether they think there is a relationship between SD and QOL, and if so, how they would describe that relationship; and d) identifying the future goals of youth and learning how they connect SD with achieving these goals.

## Methods

Ethical approval for the study was obtained from the Health Sciences Research Ethics Board, Western University, London, Ontario, Canada. A qualitative descriptive methodology with semi-structured individual interviews with youth (followed by a focus group with the same youth) was used to address the study objectives (Sandelowski 2000). This approach fits within the broader area of phenomenology which seeks to understand the ‘lived experience’ of a group of individuals by listening to their perceptions (Creswell 1998). Persons who have lived the reality being studied are considered the only legitimate source of data in phenomenology (Baker et al. 1992). Using this design, researchers ask meaning questions to reveal the essence of experience and arrive at a description of the phenomenon as experienced by the individual (Morse and Field 1995).

### Recruitment

A purposive sample of youth was recruited from a children’s rehabilitation center. Youth between 11 and 20 years of age with any of type of chronic condition for which the center provided services were considered for the study. Those with progressive conditions where life expectancy is decreased were not eligible as these conditions may have unique consequences for QOL. To be eligible, a youth also needed to be able to cognitively understand and respond to questions posed by a study interviewer.

Clinicians at the children’s center were provided with information about the study and asked to suggest clients that met inclusion criteria for the study. Potential participants were sent an information package from the center’s Quality Management Director. Those who did not return a form declining further study contact were called by a study project coordinator. If they agreed to participate, an interview date was set up.

### Data Collection and Analysis

One of two clinicians (a physical therapist or occupational therapist), each with over 30 years of experience working with children and youth with chronic conditions, carried out an in-depth 60 to 90 min interview with study participants according to a semi-structured interview guide. The interview schedule was developed by the interviewers and the study investigators. The study team had a breadth of backgrounds that contributed to an understanding of child health and well-being (i.e., education, sociology, psychology, theology, physical therapy, occupational therapy, coaching, and rehabilitation). Interviews were conducted in the privacy of participants’ homes, with one exception where the participant preferred to be interviewed at the children’s center. Parents or other family members were not present for the interviews, again with one exception where the parent remained at the youth’s request to help facilitate communication.

Upon arrival at the interview site, the interviewers obtained parent and youth written consent. All interviews were audio-taped. The semi-structured interviews began with closed-ended questions used to collect the youths’ socio-demographic and health-related information. Next, youth were asked a ‘warm-up’ question about what they liked to do for fun in their spare time. Questions were linked directly to the study objectives and included: “In this study, we want to get a better understanding about what the term ‘quality of life’ means to youth. Quality of life means different things to different people. When you think of the term quality of life what does it mean to you?”; “What kinds of things make a difference to your quality of life?”; “Can you tell me about how this makes a difference to your quality of life?” (asked for each factor identified by the youth); “When you think about self-determination what does that mean to you?”; “What things have to be in place for you to be self-determined?”; “Do you think there is a connection between self-determination and your quality of life?”; and if yes, “How do they connect?”; “What are one or two goals you have for the future?”; and “What connection is there for you between SD and achieving these goals?” Initially, questions about future goals and their connection to SD were not included in the interview schedule. However, after conducting the first two interviews, it was realized that this was an important area of inquiry that should be covered by the study. The follow-up focus group was conducted at the children’s center with the same study participants. The group session was designed to let youth further consider, reflect, and expand upon the individual interview findings. The initial categorization and thematic analysis based on the original individual interview data (described below) permitted the development of the questions for the focus group.

The clinician/interviewers used a listening/facilitating style to conduct the interviews and focus group. They were non-directive, following the participants’ lead. Affirmative comments were used to reinforce strengths of participants and build rapport and trust. They did not make assumptions about understanding what was said, instead asking for examples and clarification. The pace was slow, leaving time for participant reflection. Questions were repeated or reframed as needed. Expanding phrases were used, such as: “What else?” or “Would you like to you say more about that?” Visual cue cards and sticky notes to highlight key words during the interview were used as needed to help participants respond to questions. Careful attention was given to verbal cues to determine if probing was appropriate, or whether the participants had said all they wanted to or could say.

The recorded interviews and focus group were transcribed by a professional transcriptionist and reviewed for accuracy by the youths’ interviewers. They were imported into NVivo10 (2012) by one of the project coordinators [MN] who then completed line-by-line coding and the creation of a codebook. One of the study investigators [JM] also read all of the transcripts and checked the codebook for accuracy and comprehensiveness. The research team then began a phase where codes were collapsed and ‘meaning units’ or groupings of statements began to emerge from the data. Categories and concepts derived from clusters of meaning units were identified within each of the key question areas (i.e., meaning of QOL, factors related to QOL; meaning of SD, contributors to SD; and the connection between QOL and SD; future goals and how SD is connected with meeting those goals).

Trustworthiness of the data was established through a number of processes. Credibility was enhanced within the interview process through the interviewers’ use of techniques such as reframing, repeating, and expanding questions as required (Krefting 1991). Members of the research team had extensive experience working with youth who have chronic conditions as well as qualitative research experience (Tsonis and McDougall 2012; Hutzai et al. 2009; Gibson et al. 2012; Reid et al. 2011).

Credibility was also heightened through triangulation of the data [37]. Earlier, the interviewers [PB and JE] completed a ‘high level’ coding, where they identified overarching ideas and concepts within each interview. This was compared to overarching factors and themes identified through the process of collapsing codes and exploring in-depth meaning. In addition, a second project coordinator [NE] carried out line-by-line coding of three randomly chosen excerpts from three transcribed interviews, and the coding was compared to that of the project coordinator [MN] who originally coded the transcripts.

Finally, member checking, or checking the data with the research team’s interpretations (Krefting 1991) was carried out in two ways. Information packages were mailed to study participants summarizing the study findings, and asking them to comment on whether they were reflective of their lived experience. In addition, they were asked if there was anything else they would like to add to what had been found. Also, as noted above, the focus group provided another opportunity for participants to reflect on whether study findings realistically represented the thoughts that they had shared with the interviewers and to potentially offer any new ideas.

## Results

### Participants

The original goal of the study was to interview up to 30 children and youth, approximately 15 grade school-aged children and 15 high school-aged youth or older. However, only three school-aged children volunteered for the study, and each had difficulty understanding and responding to the questions related to defining QOL and SD. It was decided, for the purposes of the present article, to focus on analysis of the sample of 15 youth, aged 15 to 20 years (median age = 17 years). Twelve of the youth were in high school, two had finished high school, and one was attending college. Ten were male. Eight had cerebral palsy, three had another type of central nervous system disorder, and four had autism spectrum disorder, with three of these participants having Asperger’s syndrome.

### Member Checking

No new concepts or themes were emerging after interviewing the 15th youth, indicating that informational saturation had been met. The returned mailed responses from individuals and the focus group further confirmed that the youth had provided all relevant information in the interviews. During the focus group, the youth confirmed and agreed as a whole that the interview data was reflective of their lived experience. They reiterated the importance of what had been found with respect to the meaning of QOL and SD, the factors related to those concepts, the relationship between the concepts, and their goals for the future. Despite probing, no novel thoughts arose during the session. Any ideas that emerged reflected those that had been captured in the interviews and could be linked to already existing codes.

### Coding Agreement

Eighty-six percent agreement was found between the two coders on the three randomly chosen excerpts from three transcribed interviews. Where they did not agree, it was because the second coder had not assigned a code to a line. After they conferred, the second coder agreed that the code assigned by the first coder was suitable.

### Themes

The themes are presented below according to the study objectives and six key questions. The quotes that are shared reflect the dominant ideas of the participants and also highlight variation in thinking through representation of individual participants’ ideas that differed from the others. Table 2 includes specific participant responses to questions about QOL, SD, and the relationship between the two concepts.

**Table 2:** Participant responses to QOL and SD interview questions

QOL definition	Factors related to QOL	SD definition	Contributors to SD	Whether/How QOL/SD Connected
“How enriched and fulfilling your life is.”	Family, friends, recreational activities,talking to people	“You are able to be determined in everything you do… your drive to achieve.”	Interest, motivation	Yes - “You need to be determined and to reach your fulfillment.”
“How you enjoy life and get the best out of life.”	Sports teams, friends, family, school, and being independent	“Knowing you can do that and not letting other people say you can’t do that and not giving up and always trying your best.”	Guidance counselors, teachers, educational assistants	Yes - “You have more options.”
“Making sure that everyone has a good life.”	Parents, food on the table, anything can affect QOL	“To will yourself to get the things that you need to get done done.”	Self-reliance, getting things done, caring for yourself	Yes - “It sets you [up] for what you’re going to do in the future.”
“You live life to the fullest and you never give up.”	Support from friends at school, EA and resource teacher, family, doing well at school	“That one’s self should be determined in whatever they do, whether it’s sports or school or [anything].”	Understand the task, support from teachers, EAs, and friends, hands-on activities	Yes - “Because when you’re free and independent, you feel really good about accomplishing tasks.”
“To be alive.”	More wheelchair accessible places, being human, being loved	“Like I am determined to get out of school.”	Go out on your own	Yes - “Just being freer.”
“Doing the best you can with your circumstances and abilities. And being as, probably, independent as possible and relying on others but not being completely reliant on others.”	How you feel about yourself, how you come off to others, talking with others and having a good laugh, doing well in school/learning new things, being able to take the transit…gives independence	“Doing the best you can do at a task or something you want to do in life, sort of without giving up. Even if you have to do it differently than others, or it takes you longer, as long as you sort of did it yourself with, like minimal involvement of others, like not having to rely on others.”	Support for what you want to do from family, friends, and people at school, school resources	Yes - “If you have a sense of accomplishment that you’ve done things yourself, you’re probably going to have this perception that your quality of life is a lot better.”
“Waking up and feeling happy…Having a sense of purpose…also having a job that I feel serves people, or animals, or something like that.”	Having a good support team, talking with teens through similar situations, writing	“Being determined, like not backing down. Standing up for what you want, and what you need as a person.”	Self-confidence, having people believe in your goals, having people help you achieve your goals, not being afraid to ask for assistance, accessing care	Yes - “because as my self-confidence went down, my ability to be self-confident went down as well.”
“How a person lives…if they are in a wheelchair or if they can walk like you and I.”	Mom and Dad, IEP at school	Did not know	Buy a house on your own, able to keep up your own house	Yes - “Because you are able to do more things.”
“How satisfied you are with living.”	Being able to do what you like to do, a job you enjoy, friends, family, access to basic necessities, level of education, resources to go farther with learning	“Being motivated to achieve your goals.”	Motivation, interest, being able to express opinion, being able to live independently	Yes - “If I’m not motivated…I sort of stagnate I guess.”
“It makes me feel good.”	Friends, getting out, shopping, family, family dog, new walker	Did not know	Computer, accessible sink, exercises to make hand stronger, automatic garage door	Yes - “Because I like to try to be as independent as possible.”
“The value of your life and how much you think you are enjoying your life, or how good your life is for you.”	Friends, learning, parents, knowledge, health	“One’s willingness to do what they can to be all they can be. To strive through any situation, be it good, bad, ugly.”	Willpower, make your own food, pay your own rent, transportation	Yes - “You need to have self-determination in order to improve yourself and improve the circumstances around you for you to have a quality life.”
“What you do in life.”	Friends, family, a good job	“To have enough confidence to be yourself.”	Myself as a person, mother, learning from siblings	Yes - “You don’t want other people to influence who you should be.”
“To go do more better things with my life, living on my own.”	Eating healthy, grooming and hygiene, being nice to people, friends, family, eating out, nice bed to sleep in	Did not know	Making decisions and setting your mind, communicating with others	Yes - “Independency is like a number one priority for me.”
Did not know	Go see hockey, friends, help other people	Did not know	Having choices	Did not know
“Be a useful person and be happy.”	Away from parents, independence, hang out with friends, getting a high job	“I make my own choices.”	Freedom from parents, help from people, places that can help you work on getting an apartment	Yes - “I can make my own choice of what I think I’m good at.”

*QOL* quality of life, *SD* self-determination, Definitions of QOL/SD and answers to how they are connected are verbatim quotes; factors related to QOL and contributors to SD is summarized information pulled from the transcripts

1. “When you think of the term ‘quality of life’, what does it mean to you?”

#### QOL as an Overarching Personal Evaluation of Life Overall

The definitions of QOL provided by the study youth overwhelmingly indicated that they viewed this concept as an overarching personal evaluation of their life (see Table 2). For example, participants referred to it as: “How satisfied you are with living.”; “How you enjoy life and get the best out of life.”; and “How enriched and fulfilling your life is.” One youth described QOL as: “The value of your life and how much you think you are enjoying your life, or how good your life is for you.”

#### Purpose is Important to QOL

A number of the youth saw having a purpose in life as tied to their overall life quality. For example, one youth stated, “Waking up and feeling happy…Having a sense of purpose…also having a job that I feel serves people, or animals, or something like that.” Another said, QOL meant to “be a useful person and be happy.” Yet another felt that it was, “Making sure that everyone has a good life.”

#### Self-Determination is Important to QOL

Others spontaneously emphasized the importance of SD to their overall QOL. One participant stated that “you live life to the fullest and you never give up.” Another youth said, “Doing the best you can with your circumstances and abilities. And being as, probably, independent as possible and relying on others but not being completely reliant on others.” This line of thinking is explained in greater detail below in the responses to the direct question on the link between SD and QOL.

Only one youth mentioned any specific factor or aspect of life within their definition of QOL, that being related to disability/mobility: “How a person lives…if they are in a wheelchair or if they can walk like you and I.” One youth did not have a meaning for QOL, even after probes were offered.

Questions 2 and 3 “What kinds of things make a difference to your QOL?” … “Can you tell me about how this makes a difference to your quality of life?”

Regarding the things that youth reported to be related to their QOL, five overarching themes emerged: relationships; supportive environments; doing things; personal growth and moving forward, and self-understanding/acceptance of disability.

#### Relationships

Most youth mentioned family, parents, or siblings as important to their QOL. The importance of family pets was also stated. A number of youth explained that their family was helpful with respect to their disability. One youth felt his family was important “because you know, I have a disability, and need help with a lot of things.” The youth said it was also good spending time with family and learning from them…“you know, my Mom and Dad, but I can throw my siblings in there too, [be]cause, you know, they teach you to love too.” Another youth commented that family “goes out of their way, like…my Dad built me my desk, he built me my dresser…and my Dad went all the way up to…to get the [wheelchair] van.” One participant stated that his father “taught me to be very responsible…my mother she’s taught me to express myself and to be very understanding and supportive of others.” Another expressed “Having a good time together [with family].”

Some participants expressed mixed feelings about being with family and wanting to be independent. One youth said, “I’m not being myself, [when I say to my parents] I don’t want to live here”, but at the same time was frustrated about not being able to meet age-expected milestones: “I can’t get my driver’s license, my Dad said I can’t.” Another youth said more wheelchair accessible places would mean “I won’t need my Mom or Dad around every day.” One participant stated, “As long as I am away from my parents, [be]cause my Mom and my Dad always, they lecture me a lot.”

The majority of youth identified friends as being important to their QOL. As one youth explained, “I like spending quality time with people my own age.” Friends, like family were often seen as being helpful and supportive. One participant talked about how “friends give you acceptance, encouragement, someone to talk to, [and] someone to learn from.” Talking with teens in similar situations was seen as important. As one youth explained, “because then I feel like I’m not the only one that may struggle in this area, so I like it, because then I don’t feel so alone in my struggle.” Another youth named several friends and what they had taught him, and concluded, “And I believe that’s it, that’s what all my friends have taught me, to have good quality in my life.” While not a dominant comment, some expressed interest in romantic relationships. Quality of life was improved by “having someone special in your life, and I think you know what I am talking about.” said one participant. One youth said he had a girlfriend, and that it made a big difference to his QOL.

Some participants reported wanting more close friends. One youth said he would like “more friends…having their actual phone number so you can talk to them, having them over, even for 5 min is priceless.” Others experienced bullying. One youth commented, “I haven’t let harmful words bring me down because bullies, they just want someone that it harasses.” Another said, “oh there was all this bad stuff when I was 16, 17, being like harassed, rude, being spat at and all that, hit.”

The importance of people other than family or same-age friends to their QOL was described by youth. “Having a good support team that support my goals and my vision of what I want to happen in life” was identified as making a difference to QOL by one youth. Tangible and emotional support from teachers and other staff at school such as educational assistants (EA) was mentioned by several youth. One said that “just knowing that, like, my guidance counselors, teachers, EAs, like staff, are there when you need them.” Conversely, one youth was happier when he no longer needed an EA, and another was pleased when his EA was changed, “I couldn’t be myself because my EA was really boring, and that eventually leaked into my personality which wasn’t the real me.”

While youth appreciated the support they received from others, some also mentioned that “being nice to people” and “helping other people” was important to their QOL. One youth commented, “I don't think it’s enough to just be happy as a person until you’ve done something for someone else. I couldn’t be happy without knowing that I’ve made others happy as well.”

#### Supportive Environments

Youth identified several environmental aspects of the home, school, and community as making a difference to their QOL, such as availability of resources, physical accessibility and overall social supportiveness. Youth mentioned appreciating the comforts and security of home such as, “[parents] always make sure there is food on the table” and “having a nice bed to sleep in.” School resources and supports were talked about by many youth. One youth said that “it just makes school more enjoyable if you take advantage of the resources that are available.” Another participant commented that the “career planning and those supports at school” increased QOL. On the other hand, when high school was finished, he said he “lost the structure in my days…I lost…the social aspect of my life.” However, when transitions went well, the result was one of higher QOL. One youth commented that the high school environment encouraged independence. As she explained, “in public school, it seemed you were kind of told what to do, and in high school you’re on your own.” Another felt that starting university improved her QOL because she “made a lot more friends and, like, my knowledge base has increased.”

Summer camp was also mentioned by some participants as a place to gain independence as well as make new friends. One youth commented, “Those were definitely a really good rock solid support system [be]cause I had people who actually wanted me there.” However, as with school, aging out of camp “was a real wake-up call, it hit me like a ton of bricks thinking that I’m too old for this now.” Proximity to services was helpful. One participant explained that moving from a rural to an urban setting improved his QOL “because we were much closer to facilities around town.”

Having “more wheelchair accessible places” was mentioned as important to QOL by several youth. Sometimes, lack of accessibility worked in a youth’s favor, such as when one young woman went to see a play with her school classmates, “The other kids got to go on the [bus], and I couldn’t because it is not accessible, but [the train] is better.” Transportation, equipment, and medication were mentioned by several participants as improving QOL. For example, one youth said her sixteenth birthday was exciting because one of her presents was a “wheelchair van [be]cause it had a bow on it.” One youth commented that “the pill I’m taking right now…I feel is a great improvement.” However, when he was on the wrong medication, “that was terrible. It was, like it did the opposite effect.”

#### Doing Things

Youth often mentioned taking part in leisure and recreational activities as enhancing QOL. One participant enjoyed “events that are going on, you know that’s going on around the city, you know, movies, the mall, mmm, dances, even going to [the donut shop].” Another commented that she “liked the mall…shopping, getting out of my house so Mom can have her quality time.”

A number of youth brought up enjoying sports and being on sports teams or playing games as benefiting their QOL. One youth said going to the gym and playing wheelchair basketball contributed to his QOL and that, “I’m actually better at wheelchair basketball than regular basketball.” Another youth enjoyed “going to [hockey] games. Yeah, my friend has season’s tickets.” One participant stated, “It’s always fun to laugh with my friends and sometimes we all just get together and play a game Dungeons and Dragons, which is really fun [be]cause they are all very humorous about the game, but they’re also very good players.”

#### Personal Growth and Moving Forward

Learning and doing well in school was very significant to the QOL of youth. One youth explained, “For me, if I’m doing well in school, then I sort of know that in a sense I’m doing something right and I don’t know, it’s sort of proof on paper that I’m doing well in life and that sort of thing.” Another talked about his experience in a co-op: “You don’t think you’re needed at a co-op or anything. Then I realized, like, when you go to co-op you are needed and the teachers have given me more tasks because they can trust me more with it and that makes me feel important.” This youth was the first to graduate among his siblings, “and that was a really important goal to graduate from high school with honors was an extra for me. If I can do this, then I can do this and that.”

Participants saw getting a higher education as a definite stepping stone toward good QOL in future. One youth said, “I would focus on my work better if I go to college. I would work harder.” Another explained that college would make a difference in his QOL because “I’ll be able to go to like, a lot more jobs will be open to me.” The exuberance of one participant when describing his acceptance to college is telling of its importance to his QOL: “[I was] really excited especially when my Mom woke me up at 8 o’clock in the morning and said, very calmly, ‘by the way you’ve been accepted into college.’ Yeah, but that was all she had to say and I jumped out of bed and I started hooting and hollering and showed my Dad! I was happy!”

#### Self-Understanding/Acceptance of Disability

An understanding of self that was rooted in an acceptance of disability was identified by several participants as positively affecting their QOL. One youth noted, “There are some people with disabilities who think…‘why was I born like this’…You can’t feel sorry for yourself because there is always going to be somebody worse off.” Another explained, “There's people that give you a label, and there’s people that won’t touch you, but ultimately it’s not their fault, they’re just not educated, or they’re just… they’re not educated about that. Or they just think they should treat everybody in a wheelchair like that. But it’s not their fault, you can’t get mad at them for, like, that, it’s not a big deal…I’m just happy.” One participant exclaimed, “You know what? Asperger’s is actually a good disorder, did you know that? It makes you like very smart. As long as you get the [obsessive compulsive disorder] and the anxiety treated down, you will be everything positive.” Yet another said, “I know I have a disability, but I’m still able to do a lot of things.”

Question 4 “When you think about “self-determination” what does that mean to you?”

#### Independence, Freewill, Believing in Yourself

For youth, the meaning of SD was tied to a personal sense of self, independence, and freewill (see Table 2). One youth defined SD simply as, “being determined, like not backing down. Standing up for what you want, and what you need as a person.” Another stated, “I make my own choices.” One youth defined it in terms of having “enough confidence to be yourself.” Four of the participants did not have an immediate definition of SD, but when given probes, two of the youth responded as such: “I choose to play my games, food, pick my clothes.” and “Yeah, that’s what I want to do too, like moving out, out of my house.”

#### Goal Achievement

Some included goal achievement in their definition of SD. One youth said, “Being motivated to achieve your goals.” Another stated, “Like I am determined to get out of school.” Still another youth explained, “That one’s self should be determined in whatever they do, whether it is sports or school or [anything].”

#### Perseverance and Willpower in the Face of Challenges

Others described SD as perseverance and willpower in the face of challenges. One youth stated: “Doing the best you can do at a task or something you want to do in life, sort of without giving up. Even if you have to do it differently than others, or it takes you longer, as long as you sort of did it yourself with, like minimal involvement of others.” Another participant thought SD was “One’s willingness to do what they can to be all they can be. To strive through any situation, be it good, bad, ugly.”

Question 5 “What things have to be in place for you to be self-determined?”

Three general theme areas were identified by youth as contributing to their SD: personal strengths, interdependence, and functional independence.

#### Personal Strengths

Most youth described personal strengths as contributing to their SD, such as being independent, having willpower, perseverance, self-reliance, confidence, motivation, a desire to achieve, skills, and an understanding of their goals. Indeed, aspects of self were what emerged primarily in their definition of SD. One youth commented, “When I realized that the circumstances of the way I’m living are not up to what I want then I just have to have the willpower to give myself the self-determination to get through this and to find a way to improve myself and the way I live.” Another youth said, “If I want something, my mind’s set on it, I can do whatever I want, my mind’s set on it.

#### Interdependence

Although youth were clear that intrinsic aspects of themselves contributed to their SD, many were also quick to mention how other people helped them to be self-determined. Support to be able to function autonomously was seen as important. As one youth said about what needed to be in place for him to be self-determined, “probably the support of my family and friends, and them, like helping me with whatever goal that I’m doing, or just being supportive of what I want to do and not telling me that can’t do it, and the support of the people at school.” Another participant stated that “having people that believe in my goals, having people that can help me achieve them and the ability not to be afraid to ask for that assistance.” One youth felt that he had, “to be able to understand the work that needs to be accomplished, and the support from my teachers and EAs and friends.” One youth described being inspired by “other people and wanting to do that too.” “Being a valued member of making decisions” was mentioned by another participant. One youth saw helping others as important to his SD, and said about the study interview: “I want this to help people and that’s actually why I decided to do it.”

#### Functional Independence

Being able to get around on one’s own, take care of one’s self, and communicate freely with others was seen as supporting youths’ self-determination and was facilitated by accessible transportation and resources. One youth said “soon I’ll be getting a form of motor transportation that I can use myself. Not my own car, but one of those scooters, you know, with the pedals. That’d be nice to have [be]cause then it’ll get me from A to B without having my parents wake up.” Having a computer, an accessible sink, and an automatic door opener were other things mentioned by another youth. Some youth identified access to services that would facilitate independent functioning at home and in the community as contributors to SD.

Question 6 “Do you think there is a connection between SD and your QOL … and if, yes, how do they connect?”

#### SD Leads to Good QOL

All but one youth felt that there was a connection between their SD and QOL (see Table 2). One participant said, “You need to have self-determination in order to improve yourself and improve the circumstances around you for you to have a quality life.” Another explained, “If you have a sense of accomplishment that you’ve done things yourself, you’re probably going to have this perception that your quality of life is a lot better.” Yet another said, “When you’re free and independent, you feel really good about accomplishing tasks.” Participants also saw a link between SD and their future QOL. One youth commented that, “you need to be determined to reach your fulfillment.” Another said, “It sets you up for what you are going to do in the future.”

Questions 7 and 8 “What are one or two goals you have for the future?” … “What connection is there for you between SD and achieving those goals?”

#### Youth Want for Their Future What Most Youth Want

Youth identified a number of goals that they saw as contributing to good QOL in the future, including: going to college, finishing college, travelling, getting an apartment, living independently or more independently, spending time with friends, getting a job, having a career, such as being a teacher, getting married, and raising a family (see Table 3). The types of goals mentioned by participants reflected the same types of goals of most youth. Getting a job was seen as vital, and mentioned most often as a future goal. One youth said, “I’d like to get an apartment with a roommate that I can split the rent with…I would like to get a job as a baker’s apprentice, and then move up to become a baker. I’d like to spend more time with my friends…and I’d also like to go places that I enjoy going to, and having fun there.” Youth saw passing college as a key stepping stone to getting a job they liked. As one participant commented, he was looking forward to “getting through college and being able to find a job that I am happy with.”

**Table 3:** Participant goals and connection between SD and goals

Participant goals	Connection between SD and goals
Not asked	Not asked
Not asked	Not asked
Travelling, living independently	“I just stay focused on my goals. I know that if I do one thing, I’m going to have to do something else first. I know I have to get a good paying job in order to reach those goals.”
Become a teacher, raise a family	“If you’re really self-determined…and you want to achieve, and want to become a teacher, [you] have to work really hard at college.”
Work as a marketer from home	“Graduate from school.”
Finish university, move out of family home	“Willing to put effort in myself in order to complete [university], it doesn’t just sort of happen by someone else’s power.”
Accessing care to live independently	“You don’t give up and you keep going…until you find someone that sees your goal or your dream to do something on your own.”
Pass college, get a job as a child and youth worker, get married	“[Learning] how to do things.”
Getting through college, finding a job	“This is what I want to do, not what someone else wants me to do.”
Work Get an apartment, get a job in the food industry, spend time with friends, travel	“I might need help with getting ready for work.” “I need the self-determination to be a responsible young adult and eventually adult, so that I can pay for my own items and look after myself without anyone’s help.”
Get an apartment, go to college	“Because I’m self-determined, I know that it’s something that I can set out and when I want to do it and I’ll be able to get through it, persevere through anything.”
Move out of parents’ home, get a better job	“I think of a light switch, you want to do this.”
Find a job, participate in recreational activities	Did not know
Work as an architect, live independently	“I just may need some help if I don’t know how to do it, but I have to make my own choice of what I’m doing, but I don’t like people telling me to make what choices.”

*QOL* quality of life, *SD* self-determination, Participant goals are summarized information pulled from the transcripts; answers to how SD and goals are connected are verbatim quotes. Questions about goals and their connection with SD were added to the interview schedule after the first two interviews took place

### Connection Between SD and Future Goals

#### Staying Focused on key Life Goals

Youth spoke of being focused on key goals centered around life transition and moving forward. One participant explained, “I just stay focused on my goals. I know that if I do one thing, I’m going to have to do something else first.” Working hard to get an education from high school was seen as important. As one youth commented, it was important to “graduate from school.” Another said, “If you’re really self-determined…and you want to achieve, and want to become a teacher, [you] have to work really hard at college.”

#### Having Personal Goals

Youth also thought the connection between SD and future goals had to do with what they personally wanted for the future. One youth said, “this is what I want to do, not what someone else wants me to do.” Another said, “I just may need some help if I don’t know how to do it, but I have to make my own choice of what I’m doing, but I don’t like people telling me to make what choices.”

## Discussion

This qualitative research was conducted to build on other qualitative studies that have explored the factors and themes related to QOL for youth with chronic health conditions. This study was the first to specifically ask these youth what the term ‘quality of life’ meant to them. Findings overwhelmingly indicated that youth readily defined this concept as an overall subjective sense of satisfaction, enjoyment, or positivity in their life. Some tied purpose and self-determination directly to their sense of QOL.

These findings are similar to studies that included other pediatric/young adult populations and have asked the individuals to define QOL. For example, in a study of 31 adolescents aged 14 to 15 years old in the general population (Helseth and Misvaer 2010), it was in most cases described as ‘feeling good about one’s life, being satisfied with oneself, or having an overall positive attitude’ (p. 1456). In another study that included eight young adult survivors of childhood cancer, half revealed that QOL is achieved from enjoying life, while for the others, QOL meant living similarly to the average person, or having the ability to perform day-to-day tasks or manage life's problems (Tsonis and McDougall 2012). While these findings need to be replicated with other and larger groups, this initial work suggests that life quality tends to be experienced holistically. This lends some credence to those researchers who have argued that QOL is a subjective evaluation by a person of the overall degree of positivity in his/her life that can be influenced by multiple subjective and objective factors (Moons 2006; Anderson and Burckhardt 1999; McDougall et al. 2010b; Beckie and Hayduk 1997). For example, as one group of researchers (Beckie and Hayduk 1997) has posited, “It is entirely consistent to claim that QOL is both unidimensional and multiply caused” (p.23).

Moreover, these findings suggest that QOL might be best measured as a unidimensional construct in terms of individuals’ perceptions of their life quality overall, and that the factors related to QOL should also be measured distinctly, not as proxies for QOL. This would allow analyses of the relative influence of such factors on perceived QOL. A number of unidimensional measures exist that could be used for such purposes, such as single-item visual analogue scales (de Boer et al. 2004; Dijkers 2003) and multi-item scales of global perceived QOL (Dijkers 2003; Huebner et al. 2004; McDougall et al. 2013).

That some youth tied purpose and SD concepts to their definition of QOL may reflect the fact that study participants are at a time in their lives when they are on the verge of taking on the difficult challenge of transitioning to adult life and at the same time, having to cope with a serious chronic condition. It has been conjectured that sometimes age- and/or health-related problems can push certain factors to the fore (Dijkers 2003). Youth in this study consistently identified a sense of caring for others and wanting to make a positive contribution as significant to their life purpose. It would therefore seem of benefit for service providers to carefully listen to what is important and meaningful to youths’ lives as well as inquire about their sense of SD when conducting assessments and working toward enhancing the QOL for youth with chronic conditions.

In terms of the factors related to QOL, there were extensive ideas from the youth that were interviewed about this. The five overarching themes that emerged (relationships; supportive environments; doing things; personal growth and moving forward, and self-understanding/acceptance of disability) are indicative of those found in past research, especially in studies focused on QOL as opposed to HRQOL. They are reflective of the conceptual framework of QOL for youth with chronic conditions put forth by Renwick et al. (2003) that identifies three overlapping domains that contribute to QOL: ‘being’ (the child’s self-perception - understanding of self/acceptance of disability), ‘belonging’ (the child’s connections to people and places - relationships, doing things, supportive environments), and ‘becoming’ (the child’s development - personal growth and moving forward).

As in most studies of QOL (Cummins et al. 2010), ‘relationships’ was identified as a primary factor connected to QOL. Youth spoke of relationships with family, friends, peers, and others. For each type of relationship, both positive and negative aspects were revealed. Parents, siblings, and extended family members were seen as supportive, and participants appreciated their companionship as well as their tangible support with respect to disability related issues. On the other hand, when parents were viewed as overprotective and of restricting youths’ activities or personal goals, participants saw their parents as detrimental to their life quality. Families would benefit from education about how to introduce and support the development of autonomy and responsibility throughout childhood and adolescence, so that youth are prepared and families are willing to let go during the transition to adulthood (Stewart et al. 2009).

Friends were viewed by youth as most precious, and having a significant impact on QOL. Being able to do things with friends both at home and in the community was especially valued. Participating in sports such as wheelchair basketball or sledge hockey as opposed to being able to participate in typical sports was viewed positively overall, but could sometimes be seen as interfering with friendships with adolescents who did not have chronic conditions. As research has consistently shown, children and adolescents with chronic conditions often have difficulties with friendships and fitting in at school. Indeed, ‘peer relationship problems’ has been identified as the most significant mental health issue for these youth (Brossard-Racine et al. 2012, 2013). Initiatives to promote friendships and social support from peers should target change on multiple levels (personal, interpersonal, environmental). Among the initiatives that could help make a difference to the quality of peer relations for youth with chronic conditions are: i) assessments that evaluate interpersonal skills, recreational participation, and satisfaction with peer relations; ii) school initiatives that increase interpersonal contact among all youth and promote socially welcoming and supportive school environments; and iii) opportunities for youth to do things with peers at home and in the community, as well as at school (McDougall 2011). Accessible physical environments and transportation would also do much to promote the development of same-aged friendships among youth with and without chronic conditions.

The identification of personal growth and moving forward as a major contributor to QOL, and the inclusion of SD in some of the youths’ definitions of QOL, emphasizes the significance of this theme. Youth not only expressed hope and optimism; they expressed goal- and action-orientation. Desiring and working to do well at school and understanding the importance of this to future QOL further underscores the importance of the school environment as a place where youth need to feel safe, welcomed, and well supported. These study findings are mirrored by recent quantitative research that found school productivity and school belongingness and safety to be among the most significant correlates of perceived QOL for youth with chronic conditions (McDougall et al. 2014). Educators should support children and youth in their desire to do well and for independence, as opposed to making assumptions about their capabilities. Unfortunately, research indicates that few teachers carry out instruction to promote self-determination in students with chronic conditions and associated disabilities (Grigal et al. 2003; Wehmeyer et al. 2000). Similarly, models of pediatric health service delivery where responsibilities gradually shift from the service providers and parents to the young person, and continue to provide support into early adulthood would be of great benefit for enabling young people with chronic conditions to become independent, healthy, functioning adults (Gall et al. 2006).

Acceptance of disability was a clear indicator of life quality for youth, as well as the sense that they would not be defined or held back by their disability. There was a strong understanding and awareness of self that surpassed, as well as drew upon, this part of who they were. While disability was a motivator for some and insignificant for others, not one youth revealed that they felt incapable or sorry for themselves because of their condition and related disabilities. In fact, youth expressed feeling ‘unique’ and able to understand others’ prejudices. To support and further encourage youth in their quest for sense of self and personal identity, health professionals would do well to focus interventions on youths’ strengths and the exceptional nature of their lives, in addition to the traditional focus on functional needs. Also, since helping others was important to youths’ QOL, providing opportunities for youth to be support providers as well as receivers of support could do much to enhance their sense of self-efficacy.

Along with relationships, material well-being (acquirement of income, purchases, or associated concerns) tends to be the most often identified factor related to QOL in qualitative research (Cummins et al. 2010). What was curious about study findings, considering the popularity of social media among adolescents and its emphasis on ‘fame and fortune’ was that few youth talked about any aspect of this as being important to their QOL. This was the case even though most youth mentioned in the warm-up question that they used social media as one of the things they did for fun. Only one youth stated that he wanted to get a “high job…become famous…being a movie star.” Most others tended to express gratitude for what they had, and for their ‘basic needs’ being met. This could in part be due to the study focus on abstract and mature concepts. In addition, study participants were of an age where concern for ‘paying the bills’ had not yet become a reality. However, past research exploring what ‘success in life’ means to youth with cerebral palsy had similar findings with happiness being much more important to youth than money or status (King et al. 2000).

Another unexpected aspect of study findings was that, although most youth did refer to the influence of their condition/disability on their psychosocial functioning and mentioned related functional issues, such as accessibility and activity limitations, few youth identified chronic physical health issues as influencing QOL. In contrast, pain and other chronic symptoms have been identified in several past studies (Panepinto et al. 2012; Young et al. 2007; Hill et al. 2014; Squitieri et al. 2013; Vinson et al. 2010) as impacting youths’ QOL, particularly those studies focused on HRQOL. This study did not specifically inquire about the impact of the youths’ health or health condition on QOL in the individual interviews. This was simply left for the youth to identify on their own, if it was an issue for them. A few youth mentioned that an overall sense of feeling healthy and trying to be healthy by eating well was important to their QOL. It could be that if youth are not asked directly about how their chronic condition affects their QOL they tend not to focus on bodily issues. The topics of ‘health’ and ‘disability’ were directly introduced in the focus group to see if this would elicit any more of a response, but again, youth did not mention chronic physical health issues in relation to their QOL. Rather they discussed health in terms of a temporary, acute illness such as having a cold or as an overall sense of feeling healthy, and disability again in terms of psychosocial functioning, accessibility issues, and activity limitations.

Pediatric rehabilitation services have tended to focus on physical health and body function in past and have evolved toward a broader perspective (King et al. 2002). The findings of this study support this broader perspective, especially given that the youth did not focus on physical health concerns with respect to their QOL. Study findings related to how youth define QOL and the major themes that emerged suggest that youth with chronic conditions not only experience life quality holistically, but that they would also benefit from a holistic approach to service delivery. Undoubtedly, it is essential to address the physical concerns of youth with chronic conditions. However, study findings suggest that it is important for health professionals to work together with other professionals to provide integrated and coordinated care that will support the physical, functional, emotional, social, and developmental aspects of youths’ lives.

Youth overwhelmingly identified a connection between being self-determined and having good QOL. They defined SD in terms such as independence, believing in themselves, freewill, goal achievement, perseverance, and will power in the face of challenges. These concepts are reflective of the four essential characteristics of self-determination identified by Wehmeyer (1999): autonomy, self-realization, psychological empowerment, and self-regulation. As Wehmeyer and Schalock (2001) have explained, people who are self-determined cause things to change to accomplish a specific end, such as obtaining employment or independent living; and this process of agency supports optimization of QOL. Indeed, research has found SD to be positively related to both employment and independence in managing personal finances for individuals with learning and developmental disabilities, one and 3 years after graduation (Wehmeyer and Palmer 2003; Wehmeyer and Schwartz 1997). The youth in this study placed particular emphasis on perseverance and will power and identified the value of good supports in reinforcing their determination to ‘keep on keeping on’ in pursuit of their goals. They also made a clear link between being self-determined, meeting those goals, and enjoying future QOL.

The findings of this study also support Deci and Ryan’s (2000) self-determination theory, which states that satisfaction of three basic psychological needs: autonomy, competence, and relatedness, facilitates intrinsic motivation and is essential for development, performance, and well-being. Youth not only defined personal strengths, such as autonomy and competence as contributing to their SD, they also acknowledged the importance of others who provided them with emotional and tangible support toward their goals, a sense of being a part of decision-making, and a feeling of belonging and fitting in with society in general. Interdependence (Stewart et al. 2009) or connectedness with others (Anderson et al. 2000) has also been identified by a number of sources as being important to the development of SD, acknowledging that everyone needs social supports and networks to do well throughout life.

For these youth, functional independence also contributed to their SD. While young people in the general population may not find that getting around in the community, self-care, and communicating with others are major barriers to their SD, and tend to view obtaining a driver’s license as a rite of passage into adulthood, study participants indicated that these were issues that needed to be addressed in their lives. Families and school or health services transition programs would do best to support the development of SD in youth by familiarizing them with the multiple contexts (e.g., continuing education, work, recreational settings, and health and legal systems) within which they may need to be able to function on their own, as well as interact with others (Wehmeyer 1999; Eisenman 2007; Evans et al. 2006).

The types of goals study participants aspired to were the same types of goals that most youth would want to achieve in their future. Other studies have also found that most youth with chronic conditions want an education, a job, to have close relationships, and to live independently (Cussen et al. 2012; King et al. 2000). Youth spoke of how they would have to focus and work hard to reach their personal goals. Unfortunately, in seeking to attain their goals, these youth may face obstacles that other adolescents do not. It should, therefore, be society’s goals to provide the necessary support and to change attitudes and environments so that youth with chronic conditions have the opportunity to develop their SD, attain their broad life goals, and ultimately enjoy good future QOL.

This study was limited by the use of a small sample from a single children’s rehabilitation centre that included a restricted age range and few types of chronic conditions. Generalizability across geographic regions, ages, and conditions would have been more likely if the sample had been acquired from multiple sites, larger, and included elementary school-aged children and youth with a larger variety of conditions. However, as our interviews with three younger children suggested, inquiring about the meaning of abstract concepts such as QOL and SD may not be a developmentally appropriate line of questioning for that age group. In addition, the limited age range in this work may have allowed saturation to be met more quickly and may have made our findings more relevant to transition-age youth. Future research should include larger samples drawn from multiple geographic regions. Moreover, alternative approaches to data collection might be tried with younger children or with youth who are unable to cognitively participate in an interview or focus group. It may also be of benefit for future research to obtain information on this topic from other sources, such as family members or service providers. An external perspective complementing the subjective experiences could help to triangulate the data collected from the youth perspective. Also, future research may want to delve into youths’ aspirations for particular types of jobs in order to gather valuable insights for career guidance or counseling in schools. This study conducted a single interview with youth at a point in their life when they were beginning to transition into adulthood. It would be of great interest to interview these youth again in their early adulthood to see to what extent their SD had affected their future QOL.

## Conclusions

The group of youth with chronic health conditions included in this research viewed their QOL as a personal evaluation of their overall life, as opposed to defining it in terms of various ‘things’ or distinct domains of life. Researchers and clinicians may want to consider this finding when constructing or choosing measurement tools of QOL for use in studies or in clinical assessment. The themes related to youth QOL were similar to those found in past research and indicate the significance of relationships, doing things, the environment, personal growth and moving forward, and understanding self/acceptance of disability. SD was indicated to be important to youth’s QOL, and personal strengths, interdependence and functional independence were identified as related themes. Youth identified goals for the future that are similar to those of most youth and saw SD as being essential for reaching those goals. Exploration of both subjective global QOL and SD by health and other professionals to open a dialogue about these linked concepts, and the factors related to them could promote an effective support plan to help transition-aged youth to realize their goals. Overall, the findings of this research indicate that youth have definite goals and aspirations and they recognize the need for self-understanding, independence, and ability, as well as the support of others and the environment for them to be self-determined and enjoy good present and future QOL. Service providers and decision makers may want to utilize these findings and consider a holistic approach to service delivery when developing and carrying out SD and transition interventions where the goal is to ultimately optimize QOL for youth with chronic health conditions.
